# Range Camera Self-Calibration Based on Integrated Bundle Adjustment via Joint Setup with a 2D Digital Camera

**DOI:** 10.3390/s110908721

**Published:** 2011-09-08

**Authors:** Mozhdeh Shahbazi, Saeid Homayouni, Mohammad Saadatseresht, Mehran Sattari

**Affiliations:** 1 Department of Geomatics Engineering, University of Tehran, North Amriabad Street, Tehran 11155-4563, Iran; E-Mails: homayounis@ut.ac.ir (S.H.); msaadat@ut.ac.ir (M.S.); 2 Department of Geomatics Engineering, University of Isfahan, HezarJerib Street, Isfahan 81746-73441, Iran; E-Mail: sattari@eng.ui.ac.ir

**Keywords:** PMD range camera, integrated self-calibration, bundle adjustment, joint setup, digital camera, internal error, range systematic error

## Abstract

Time-of-flight cameras, based on Photonic Mixer Device (PMD) technology, are capable of measuring distances to objects at high frame rates, however, the measured ranges and the intensity data contain systematic errors that need to be corrected. In this paper, a new integrated range camera self-calibration method via joint setup with a digital (RGB) camera is presented. This method can simultaneously estimate the systematic range error parameters as well as the interior and external orientation parameters of the camera. The calibration approach is based on photogrammetric bundle adjustment of observation equations originating from collinearity condition and a range errors model. Addition of a digital camera to the calibration process overcomes the limitations of small field of view and low pixel resolution of the range camera. The tests are performed on a dataset captured by a PMD[vision]-O3 camera from a multi-resolution test field of high contrast targets. An average improvement of 83% in RMS of range error and 72% in RMS of coordinate residual, over that achieved with basic calibration, was realized in an independent accuracy assessment. Our proposed calibration method also achieved 25% and 36% improvement on RMS of range error and coordinate residual, respectively, over that obtained by integrated calibration of the single PMD camera.

## 1. Introduction

Providing three-dimensional information about the real environment is an essential factor for various applications in industry, computer vision, automation, multimedia, robotics, mobile mapping and many more fields. In recent years, Time-of-Flight (ToF) devices, based on Photonic Mixer Device (PMD) technology, are becoming increasingly popular solutions in 3D imaging applications [1–4]. Known as range cameras, this new generation of active devices can capture range, amplitude and intensity images simultaneously with one array sensor at video rates. On the range image, also called depth image, each pixel individually measures the distance to the observed point by computing the turnaround time of the modulated near infrared light. The amplitude image shows the signal strength of the active illumination, while the intensity image represents the gray-scale values of pixels.

The main advantages of range cameras in comparison with traditional 3D data acquisition systems such as laser scanners or stereo cameras are as follows:

- No scanning mechanism is required,
- Only one sensor is needed to capture 3D data without getting involved in different stereo analysis problems, and
- Rapid imaging at a high frame rate provides the possibility of real time mapping and localization.

Due to several systematic error sources, however, proper calibration of such cameras is obligatory in order to perform reliable range sensing [5–23]. The range error sources will be discussed in Section 3. There exist a variety of studies focusing on metric performance of range cameras and developing calibration methods. Several works have studied the application of standard camera calibration procedures to estimate lateral calibration parameters of range cameras [6–8]. Range systematic errors of these devices have also been handled in different ways. Kahlmann *et al.* applied look-up-tables to adjust range measurement errors [9]. Their work involved calibrating the camera using known distances. The camera, however, had to be manually fixed at pre-located positions in such works. On the other hand, other researches considered parameter modeling of the observed error on measured ranges. Lindner and Kolb provided a B-spline approximation for range errors in [10,11], adding a CCD camera for the latter. They also improved their results by considering intensity related errors in range measurements. Fuchs and Hirzinger utilized a robotic arm to position the range camera as well as a higher-accuracy laser scanner to perform the range calibration in [12]. Schiller *et al.* proposed calibration of a PMD camera using a checkerboard pattern together with a multi-camera setup [13]. In spite of having various benefits over single camera calibration, their work still performed a two-step controlled calibration procedure. Linder *et al.* also proposed a calibration technique, based on Analysis-by-Synthesis [14]. Their method determines the intrinsic and external parameters of a ToF camera, combined with CCD cameras to synthesize the depth images. The distance and reflectivity related errors on range observations are then estimated one after another. Calibrating a single ToF camera suffers from a high dependency between intrinsic parameters and camera position due to its small field of view (FoV), which can be overcome with an additional CCD camera as discussed by [11,13,14].

Karel was the first to consider self-calibration of range cameras to overcome the problems with instability in camera parameters [15]. This method first calibrates the lateral parameters using a pre-built photogrammetric program system [16], and then evaluates the different parameters affecting the range distortion. Robbins *et al.* [17], Chiabrando *et al.* [18] and Pattinson [19] also performed a two-step calibration procedure, *i.e.*, photogrammetric calibration of amplitude images followed by separate distance error evaluation. Integrated self-calibration of range cameras which allowed simultaneous calibration of the camera lens and rangefinder systems was proposed in [20,21]. Neither of these works, however, considered the effect of intensity dependent errors on range measurements. In their integrated approach [22], Westfeld *et al.* performed photogrammetric calibration of a range camera considering additional distance error terms. Lichti *et al.* conducted an experiment to compare the performance of three range camera self-calibration methods: the one-step integrated; the two-step dependent; and the two-step independent [23].

This article presents an integrated range camera self-calibration method utilizing a low-cost custom-made system, composed of a PMD range camera (PMD[vision]-O3) and a digital camera (Canon Power Shot SX1 IS). Rigid combination of cameras and estimation of the PMD camera positions relative to the RGB camera along with integration of the range calibration into the photogrammetric bundle adjustment of the intrinsic and external parameters are the main characteristics of this work. It is suggested that this method can overcome the problems inherent to the small FoV and low resolution of the range camera. It is also assumed that the method can provide optimum estimates of the model parameters by incorporating the systematic errors and external parameters into the bundle adjustment based on weighted least squares criteria.

The following parameters are simultaneously estimated as the outcomes of the calibration process: interior orientation, radial and decentring lens distortion, sensor affinity and shear parameters, and also circular distance related error, signal propagation delay error and intensity related error parameters on range observations.

In the following section the operation principles of range imaging sensors based on PMD technology are briefly explained. Then, the geometric models are thoroughly described in Section 3. The details of calibration experiments including network and test field design are represented in Section 4. Section 5 reports the results of calibration method in terms of parameters accuracy and correlations, effectiveness of the systematic error models and noticeable impact of joint calibration with RBG camera. Finally, conclusions and future work are discussed in Section 6.

## 2. Range Imaging Principles

Range cameras based on PMD technology operate on the Time of Flight (ToF) concept to provide distance information. Typically, a PMD camera consists of a PMD chip and its peripheral electronics, emitter and receiver optics and other standard camera parts. The emitter, which is an illumination source, emits near infrared light. The reflected light is then received to measure the distance to the object. In contrast to typical ToF devices, e.g., laser scanners, all pixels in the PMD array simultaneously analyze the received optical signal to measure the depths of the corresponding points in space. The PMD chip is based on CMOS technology, which also provides an automatic suppression of background light, allowing the device to be utilized outdoor as well as indoor [24].

**Figure 1.**
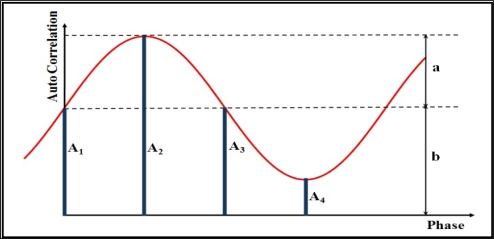
Autocorrelation function, phase shift, amplitude and intensity.

In order to provide range information, a reference electrical signal is applied to the modulation gates of each pixel on PMD array. Additionally, the incident light on photo-gates of pixels generates a second signal. The received optical signal differs from the reference one by a phase shift proportional to the depth of the reflecting target. To calculate the distance, the autocorrelation function of electrical and optical signal is analyzed by phase-shift algorithm (Figure 1). Using four samples A1, A2, A3 and A4 each shifted by 90 degrees the phase shift, which is proportional to the distance, can be calculated using the following equation:

(1) $$\varphi =\text{arctan}\left(\frac{A_{1}-A_{3}}{A_{2}-A_{4}}\right)$$

In addition to the phase shift, two other values are extracted; signal strength of the received signal (amplitude) and the offset *b* of the samples which represents the gray-scale value of each pixel (intensity):

(2) $$a=\frac{\sqrt{{\left(A_{1}-A_{3}\right)}^{2}+{\left(A_{2}-A_{4}\right)}^{2}}}{2}$$

(3) $$b=\frac{A_{1}+A_{2}+A_{3}+A_{4}}{4}$$

The distance *d* to the target is therefore given by Equation (4):

(4) $$d=\frac{c.\varphi }{4\pi .f_{\text{mod}}}$$

where *c* is the speed of light and *f*_mod_ is the modulation frequency of the emitted signal [24].

## 3. Calibration Model

In the following subsections, the models used to calibrate range and lateral parameters of PMD camera in combination with digital camera are described. Initially, the procedures that have to be executed once before the main range camera calibration are explained. This is followed by a description of the imaging geometry of two cameras, systematic error models and integrated bundle adjustment process.

### 3.1. Pre-Calibration Process

As described in Section 1, a joint setup is used to calibrate the range camera. It contains a digital camera and a PMD range camera. In order to get more accurate results, individual photogrammetric calibration of the RGB camera has to be performed before the main process. It is worth noting that the calibration process of the digital camera is done only once and its results are applied as inputs to the main calibration process of the range camera. The specifications of the RGB camera used in this work are listed in Section 4.

Calibration of the digital camera in this study is performed by the conventional photogrammetric calibration method, a computational method whereby camera parameters are estimated in a bundle adjustment solution [25]. The process uses collinearity equations that have been augmented with additional terms to account for adjustment of principal distance, principal point coordinate, systematic radial and decentering lens distortion. The equations are more described in Sections 3.2 and 3.3. The same test field of range camera calibration in a free network adjustment can be applied. However, another test field which is particularly designed for digital camera calibration is utilized. As calibration of digital cameras is a well-explored and traditional topic in digital photogrammetry, it is not discussed here in details. For more detailed explanation, refer to [25,26]. The calibration parameters of the digital camera are reported in Section 5.

### 3.2. Imaging Geometry

**Figure 2.**
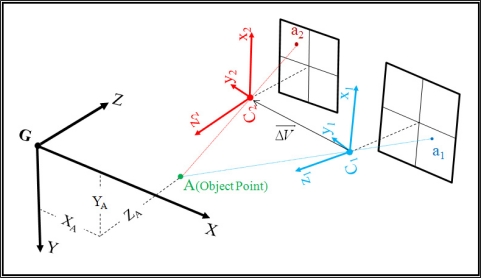
Coordinate systems and their relationships.

In this study, orientation of the PMD camera is estimated relative to the digital camera. This means that there would be six parameters of exterior orientation at each imaging station for the digital camera and only six parameters of relative orientation at all stations for the range camera. Figure 2 illustrates the relationship of the coordinate systems considered in the task: *C_1_* is the perspective center and origin of coordinate system of the digital camera, *C_2_* is the perspective center and origin of PMD coordinate system and *G* is the origin of object space coordinate system.

Assume that (*ω_1_*, *φ_1_*, *κ_1_*) are orientation angles of the RGB camera system in relation with object coordinate system, respectively around *x*, *y* and *z* axis, $\left(X_{1}^{C},Y_{1}^{C},Z_{1}^{C}\right)$ are the object space coordinates of point *C_1_*, (*ω_2_*, *φ_2_*, *κ_2_*) are orientation angles of PMD coordinate system in relation with the digital camera system and $\vec{\Delta V}=\left(\Delta X,\Delta Y,\Delta Z\right)$ is coordinate vector of point *C_2_* in the digital camera coordinate system.

Therefore, the object space coordinates of point *C_2_* would be:

(5) $$\left[\begin{array}{c} X_{2}^{C}\\ Y_{2}^{C}\\ Z_{2}^{C} \end{array}\right]=\left[\begin{array}{c} X_{1}^{C}\\ Y_{1}^{C}\\ Z_{1}^{C} \end{array}\right]+(R_{1}\left(\omega_{1}\right).R_{2}\left(\varphi_{1}\right).R_{3}(\kappa_{1}))\left[\begin{array}{c} \Delta X\\ \Delta Y\\ \Delta Z \end{array}\right]$$

where *R_1_*, *R_2_* and *R_3_* are the fundamental rotation matrices.

The observations equations for range camera bundle adjustment logically stem from three kinds of observations:

1. Two image point collinearity equations for each pixel, which is the projection of an observed target on the digital camera image.
2. Two image point collinearity equations for the pixel on the intensity image captured by range camera.
3. One range equation for the pixel on the range image captured by range camera.

For object point *i*, observed in image *j* of digital camera (indexed by “1”), two collinearity equations can be written:

(6) $$\begin{array}{l} \left(x_{\mathrm{ij},1}-x_{p1}\right)+{\Delta x}_{\mathrm{ij},1}+f_{1}\frac{U_{\mathrm{ij},1}}{W_{\mathrm{ij},1}}=0+ɛ\\ \left(y_{\mathrm{ij},1}-y_{p1}\right)+{\Delta y}_{\mathrm{ij},1}+f_{1}\frac{V_{\mathrm{ij},1}}{W_{\mathrm{ij},1}}=0+ɛ \end{array}$$

where:

(7) $$\left[\begin{array}{c} U_{\mathrm{ij},1}\\ V_{\mathrm{ij},1}\\ W_{\mathrm{ij},1} \end{array}\right]=R_{3}\left(\kappa_{j,1}\right).R_{2}\left(\varphi_{j,1}\right).R_{1}\left(\omega_{j,1}\right)\left[\begin{array}{c} X_{i}-X_{j,1}^{C}\\ Y_{i}-Y_{j,1}^{C}\\ Z_{i}-Z_{j,1}^{C} \end{array}\right]$$

in which (*x_ij,1_*, *y_ij,1_*) are image coordinates of observations on the digital camera, (*X_i_*, *Y_i_*, *Z_i_*) are the object space coordinates of point *i*, $\left(\omega_{j,1},\varphi_{j,1},\kappa_{j,1},X_{j,1}^{C},Y_{j,1}^{C},Z_{j,1}^{C}\right)$ are exterior orientation parameters of image *j*, (*x_p1_*, *y_p1_*, *f_1_*) are principal point coordinates and principal distance called interior orientation parameters and (*Δx_ij,1_*, *Δy_ij,1_*) are systematic errors of the digital camera which are known from its single calibration process and will be described in the next subsection.

There will be two collinearity equations for object point *i*, observed in image *j* of PMD camera (indexed by “2”):

(8) $$\begin{array}{l} \left(x_{\mathrm{ij},2}-x_{p2}\right)+{\Delta x}_{\mathrm{ij},2}+f_{2}\frac{U_{\mathrm{ij},2}}{W_{\mathrm{ij},2}}=0+ɛ\\ \left(y_{\mathrm{ij},2}-y_{p2}\right)+{\Delta y}_{\mathrm{ij},2}+f_{2}\frac{V_{\mathrm{ij},2}}{W_{\mathrm{ij},2}}=0+ɛ \end{array}$$

as well as one range equation as follows:

(9) $$\sqrt{{\left(X_{i}-X_{j,2}^{C}\right)}^{2}+{\left(Y_{i}-Y_{j,2}^{C}\right)}^{2}+{\left(Z_{i}-Z_{j,2}^{C}\right)}^{2}}-\rho_{\mathrm{ij}}-{\Delta \rho }_{\mathrm{ij}}=0+ɛ$$

where $\left(X_{j,2}^{C},Y_{j,2}^{C},Z_{j,2}^{C}\right)$ are the perspective centre coordinates of PMD image *j* and can be derived based on Equation (5):

(10) $$\left[\begin{array}{c} X_{j,2}^{C}\\ Y_{j,2}^{C}\\ Z_{j,2}^{C} \end{array}\right]=\left[\begin{array}{c} X_{j,1}^{C}\\ Y_{j,1}^{C}\\ Z_{j,1}^{C} \end{array}\right]+\left(R_{1}\left(\omega_{j,1}\right).R_{2}\left(\varphi_{j,1}\right).R_{3}\left(\kappa_{j,1}\right)\right)\left[\begin{array}{c} \Delta X\\ \Delta Y\\ \Delta Z \end{array}\right]$$

Therefore (*U_ij,2_*, *V_ij,2_*, *W_ij,2_*) are determined via the following equation:

(11) $$\left[\begin{array}{c} U_{\mathrm{ij},2}\\ V_{\mathrm{ij},2}\\ W_{\mathrm{ij},2} \end{array}\right]=R_{1}\left(\omega_{2}\right).R_{2}\left(\varphi_{2}\right).R_{3}\left(\kappa_{2}\right).R_{3}\left(\kappa_{j,1}\right).R_{2}\left(\varphi_{j,1}\right).R_{1}\left(\omega_{j,1}\right).\left[\begin{array}{c} X_{i}-X_{j,2}^{C}\\ Y_{i}-Y_{j,2}^{C}\\ Z_{i}-Z_{j,2}^{C} \end{array}\right]$$

In the above equations, (*x_ij,2_*, *y_ij,2_*) are image coordinates of observations on PMD intensity image, (*ω_2_*, *φ_2_*, *κ_2_*, *ΔX*, *ΔY*, *ΔZ*) are relative orientation parameters of PMD camera unique to all images, (*x_p2_*, *y_p2_*, *f_2_*) are interior orientation parameters, *ρ_ij_* is the measured distance from perspective center to object *i* and (*Δx_ij,2_*, *Δy_ij,2_*, *Δρ_ij_*) are systematic errors which are unknown. In all the equations *ε* terms indicate respective random errors.

### 3.3. Systematic Error Models

The lateral camera systematic error model used for range camera calibration is same as the standard photogrammetric model for digital cameras [26]. Let *n* be the index of camera, *1* for the digital camera and *2* for PMD camera. Therefore the image coordinates systematic errors are:

(12) $$\begin{array}{ll} \Delta x_{\mathrm{ij},n} & =\left(x_{\mathrm{ij},n}-x_{p,n}\right).\left(K_{1,n}r_{\mathrm{ij},n}^{2}+K_{2,n}r_{\mathrm{ij},n}^{4}+\ldots \right)+P_{1,n}\left(r_{\mathrm{ij},n}^{2}+2{\left(x_{\mathrm{ij},n}-x_{p,n}\right)}^{2}\right)\\ & \;\;\;+2P_{2,n}\left(x_{\mathrm{ij},n}-x_{p,n}\right).\left(y_{\mathrm{ij},n}-y_{p,n}\right)+A_{1,n}\left(x_{\mathrm{ij},k}-x_{p,n}\right)+B_{1,n}\left(y_{\mathrm{ij},n}-y_{p,n}\right) \end{array}$$

(13) $$\begin{array}{ll} \Delta y_{\mathrm{ij},n} & =\left(y_{\mathrm{ij},n}-y_{p,n}\right).\left(K_{1,n}r_{\mathrm{ij},n}^{2}+K_{2,n}r_{\mathrm{ij},n}^{4}+\ldots \right)+P_{2,n}\left(r_{\mathrm{ij},n}^{2}+2{\left(y_{\mathrm{ij},n}-y_{p,n}\right)}^{2}\right)\\ & \;\;\;+2P_{1,n}\left(x_{\mathrm{ij},n}-x_{p,n}\right).\left(y_{\mathrm{ij},n}-y_{p,n}\right) \end{array}$$

where (*K_1,n_*, *K_2,n_*, *…*) are the radial lens distortion coefficients, (*P_1,n_*, *P_2,n_*) are the decentring distortion terms and (*A_1,n_*, *B_1,n_*) are the electronic biases, namely affinity and shear, all for sensor *n*.

The range error model for PMD camera used in this study is a combination of models presented by Fuchs and May [27], Lichti *et al.* [21] and Lindner *et al.* [14] with slight differences. The model takes three main systematic range error sources into account; circular distance related error, signal propagation delay error and intensity dependent error:

(14) $${\Delta \rho }_{\mathrm{ij}}=c_{0}+c_{1}.\rho_{\mathrm{ij}}+c_{2}.{\left(\rho_{\mathrm{ij}}\right)}^{2}+c_{3}.{\left(\rho_{\mathrm{ij}}\right)}^{3}+c_{4}.\left(R_{\mathrm{ij}}\right)+c_{5}.\left(C_{\mathrm{ij}}\right)+\left(c_{6}+c_{7}.I_{\mathrm{ij}}+c_{8}.{\left(I_{\mathrm{ij}}\right)}^{2}\right)$$

In Equation (14), *c*_0_ is the rangefinder offset [21] and *c_1_*,*c_2_*,*c_3_* distance related terms [27]. Instead of a sinusoidal-base function, the circular error is modeled by a third order polynomial following the approach of [27]. Terms *c_4_*,*c_5_* are the signal propagation delay [27] also known as the clock-skew error terms [28]. Variables *R_ij_* and *C_ij_* are row and column of pixel *i* in image *j* respectively. Finally, *c_6_*,*c_7_*,*c_8_* are intensity related error terms and *I_ij_* is the gray scale value of pixel *i* in intensity image *j* of PMD camera. Since the error of the measured distance is also related to intensity, pixels with lower reflectivity tend to drift closer to the camera [14]. Following Lindner *et al.* proposed model which comprised a third order polynomial of normalized intensity [14], a polynomial function of intensity is assessed to model this error. In order to avoid over-parameterization, a polynomial of second order is used. The results show that the second order term is so small that no more terms are required. The effect of adding the intensity related terms to range error model is also investigated in Section 5.

### 3.4. Integrated Bundle Adjustment

All the unknown parameters of integrated self-calibration are simultaneously estimated in combined method of least squares sense. Observations comprise the target image coordinates and range measurements. The image measurements are made at the corners of the squares on the designed test field. However, the corners are not good references to investigate intensity related range measurement errors. Therefore, only *c_0_–c_5_* parameters are inserted into the model and *c_6_*,*c_7_*,*c_8_* will be estimated following the bundle adjustment by using the centers of squares as reference targets. This makes the terms more reliable since they have explicit intensity values in spite of the corners. Consequently, the unknown parameters in the bundle adjustment include: exterior orientation parameters of each digital camera station, six parameters of relative orientation, interior orientation, lateral systematic error terms and range error parameters (*c_0_–c_5_*) of the PMD camera.

Since the number of observations is more than that of the unknowns, a least squares solution is used. The mathematical model of observation equations expresses the relationship between observation and unknown vectors, *L⃗* and *X⃗* [29]:

(15) $$F\left(\vec{X},\vec{L}\right)=0$$

The vector function ***F*** represents equations of observations. Since the equations are nonlinear regarding the unknowns and observations, linearization is accomplished by replacing the nonlinear functions by their Taylor series approximation [24]. That is:

(16) $$F\left(\vec{X},\vec{L}\right)=F\left({\vec{X}}^{0},\vec{L}\right)+\frac{\partial F}{\partial \vec{X}}\delta \vec{X}+\frac{\partial F}{\partial \vec{L}}\delta \vec{L}=0$$

Renaming the above matrices leads to:

(17) $$W+A.\delta \vec{X}+B.\vec{V}=0$$

where *W* is called mis-closure vector and *A* and *B* are design matrices.

The least squares estimation of unknown and residual vectors is obtained by the following equations:

(18) $$\delta \hat{\vec{X}}=-{\left(A^{T}{\left({\mathrm{BP}}^{-1}B^{T}\right)}^{-1}A\right)}^{-1}A^{T}{\left({\mathrm{BP}}^{-1}B^{T}\right)}^{-1}W$$

(19) $$\hat{\vec{V}}=-P^{-1}B^{T}{\left({\mathrm{BP}}^{-1}B^{T}\right)}^{-1}W$$

where *P* is the weight matrix of observations which is initially set equal to the inverse of the covariance matrix of the observations, *∑L*.

These solutions, *δX⃗* and *V⃗*, must be added to the initial approximation of unknowns and measured observations respectively, to improve the solutions for next iteration. The iterations are repeated recursively until a convergent solution is reached. The covariance matrix of unknown parameters is defined as follows:

(20) $$\sum_{\hat{\vec{X}}}={\left(A^{T}{\left(B\sum_{L}B^{T}\right)}^{-1}A\right)}^{-1}$$

To optimize the weighting of observations with different noise levels, robust estimation by adaptive weight determination is applied in this study [30]. Instead of the initial weight matrix *P*, the equivalent weight matrix *P^V^* is determined by iterative weight functions. The weight function is based on an *a posteriori* method:

(21) $$P_{i}^{v+1}=\{\begin{array}{ll} P_{i}^{v} & \text{if }T_{i}<F_{1-\alpha ,1,r}\\ \frac{P_{i}^{v}}{T_{i}} & \text{if }T_{i}\geq F_{1-\alpha ,1,r} \end{array}$$

where:

(22) $$T_{i}=\frac{P_{i}\cdot {\hat{\vec{V}}}_{i}^{2}}{R_{\mathrm{ii}}\cdot {\hat{\sigma }}_{0}^{2}}$$

(23) $$R_{\mathrm{ii}}={\left(I-A{\left(A^{T}{\left({\mathrm{BP}}^{-1}B^{T}\right)}^{-1}A\right)}^{-1}A^{T}P\right)}_{\mathrm{ii}}$$

and *F_1-α,1,r_* is the value of F-test at the given level of confidence *α* and *r* is the number of degrees of freedom. The parameter ${\hat{\sigma }}_{0}^{2}$ is the estimate of unit weight variance. Since the combined method of least squares estimation is a well known process, no more discussion is presented on this issue. For more information on the details, readers are referred to [29].

## 4. Experiments

The following subsections provide essential information on experimental aspects of the study. First, the camera specifications and their joint setup are represented. The target field used for the calibration procedure and the network designed for the purpose are described accordingly. Finally, the method of automatic target extraction from intensity images is explained in the last subsection.

### 4.1. Camera Setup

As mentioned in previous sections, the calibration approach utilizes a PMD[vision]-O3 range camera and a Canon Power Shot SX1 IS digital camera. More specifications of each sensor are listed in Tables 1 and 2. The PMD camera is allowed to warm up for at least an hour prior to data acquisition to ensure it had reached internal temperature stability [21].

**Figure 3.**
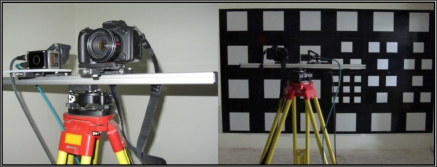
The joint setup of PMD and RGB cameras.

Cameras are rigidly mounted on a tripod as shown in Figure 3. For each imaging station, 25 frames of the PMD video are averaged to form the final image of that station. Data are acquired by the standard software supplied for the PMD camera which automatically removes low amplitude bad pixels.

### 4.2. Test Field and Network Design

**Figure 4.**
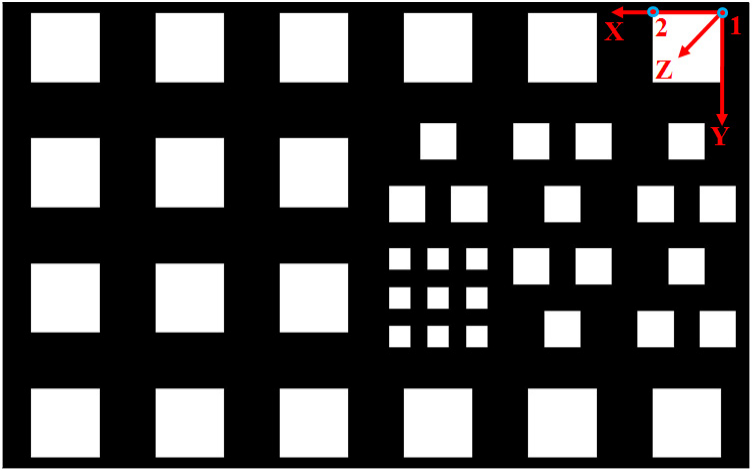
The self-calibration target field.

The test field consists of 24 multi resolution white squares on a black background rigidly attached to a flat wall, as indicated in Figure 4. Main targets are the corners of these squares. Considering the low resolution range camera, it was decided that the test field be composed of square targets because it would be possible to detect the corners of high contrast squares of proper size on PMD images. The test field dimension is 3,000 × 1,860 mm, which contains squares with three different sizes; squares of 90, 150 and 280 mm length to be detected well on PMD images at distances below 1.2, 2 and 4 m respectively. The squares are arranged in a way that there would be enough targets at every image of the network with different scales.

**Figure 5.**
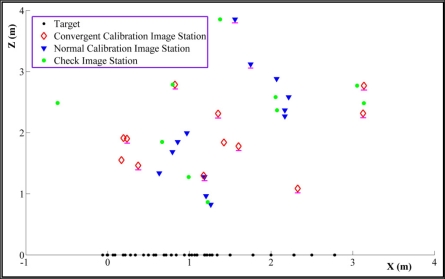
The self-calibration network and check images for independent accuracy assessment.

The network is configured to include two image sets [21]. It comprises a set of normal images (*i.e*., the range camera’s optical axis is approximately orthogonal to the target plane) at different distances, starting at 0.5 m from the test field. It should be pointed out that only the wall on which the test field is pasted appears in range images, even at the longest distance of 4 m. This set of images provides a redundant set of range observations, unaffected by scattering and multipath reflections. Both image point coordinates and range observations of these images are involved in the self-calibration adjustment. The network also includes a set of convergent images captured from several stations with enough camera rotation diversity. Only the image coordinate observations of this set are included in the self-calibration adjustment. Locations of the calibration images and object points are depicted in Figure 5. There are totally 35 exposure stations; 12 convergent calibration images, 13 normal calibration images and also 10 additional images, also called check ones, used for independent accuracy assessment.

**Figure 6.**
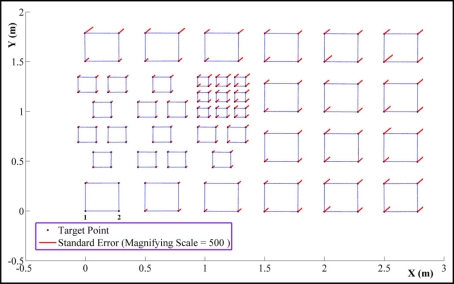
Target coordinates in object space.

In order to simplify the range camera self calibration process, the object coordinates of the targets (corners of the squares) are determined prior to main calibration process. The procedure utilizes a few (11) images of the digital camera, considering that calibration parameters of the digital camera have been determined in a pre-calibration process. These image stations are highlighted by magenta color in Figure 5. Object space coordinates of the targets are estimated in a parametric-model, network adjustment with minimum datum constraints imposed on the object points [25]. The considered right handed object coordinate system is drawn in Figure 4. The wall surface is considered as the *X*,*Y* plane and the *Z* axis points toward outside the test field plane. The origin of the coordinate system is fixed on point 1 while the *X* axis passes through point 2. The straight distance from point 1 to point 2 is measured manually to define the network true scale. The target coordinates and their standard error vectors are plotted in Figure 6.

It is important to clarify that after determining the object space coordinates of targets, they are used as pseudo observations in the main integrated calibration with their standard deviations as their weights.

### 4.3. Automatic Target Detection

The fundamental step in all the processes mentioned so far is correct detection of observed targets in intensity images. The targets are corners of white squares on the black background of the test field. In order to accomplish the task automatically and more accurate, the programs developed by Rufli *et al.* are used [31]. Briefly, this is an automatic method for detection of checkerboard corners on blurred and distorted images which best suits the PMD images as well as standard digital images. It is performed by applying binary thresholding and morphological erosion followed by a binary contour finder. Linking the right perpendicular edges leads to find the corners of a square. Since the target field of this study is not exactly a checkerboard, all corners have to be identified very approximately once. Then a patch of the image around each approximate point is put into the automatic detection program and accurate coordinates of the corner is computed. As reported by [31], this method leads to average corner inaccuracy of 0.62–1.05 pixel.

## 5. Results and Analysis

The PMD[vision]-O3 additional parameters and range error terms are determined using the procedures described in Sections 3 and 4. The algorithms are executed via the programs written particular to this approach. They can be applied to the data acquired with the standard software supplied with the PMD camera. The additional parameters of the digital camera are determined at the pre-calibration process described in Section 3.1. The results are reported in Table 3.

Through the integrated calibration of range camera based on the joint setup, the internal parameters of the camera, including lateral parameters and range error terms, are reported in Table 4. All the parameters have already been described in Section 3. The statistic F-test at the confidence level of 95% is applied to systematic error parameters to determine their significance in the bundle adjustment and ensure that the proposed model is not over parameterized [32].

In order to evaluate the test results, additional images are captured and the calibration parameters are applied to their observations. The check observations are made at the centers of the target squares. Differences of the corrected image coordinates and range observations from their true expected values are called residuals henceforth. For point *i* in image *j* of the PMD camera, the residuals are computed as follows:

(24) $$\begin{array}{l} {\mathrm{dx}}_{i,j}=\left(x_{\mathrm{ij},2}-x_{p2}+{\Delta x}_{i,j}\right)+\left(f_{2}\frac{U_{\mathrm{ij},2}}{W_{\mathrm{ij},2}}\right)\\ {\mathrm{dy}}_{i,j}=\left(y_{\mathrm{ij},2}-y_{p2}+{\Delta y}_{i,j}\right)+\left(f_{2}\frac{V_{\mathrm{ij},2}}{W_{\mathrm{ij},2}}\right) \end{array}$$

(25) $${d\rho }_{\mathrm{ij}}=-\left(\rho_{\mathrm{ij}}+{\Delta \rho }_{\mathrm{ij}}\right)+\left(\sqrt{{\left(X_{i}-X_{j,2}^{C}\right)}^{2}+{\left(Y_{i}-Y_{j,2}^{C}\right)}^{2}+{\left(Z_{i}-Z_{j,2}^{C}\right)}^{2}}\right)$$

The first parentheses of both equations correct the observations by the estimated calibration parameters. The second parentheses are the true values of the observations that are computed directly from object coordinates and camera orientation parameters, as derived in Section 3. Since the object coordinates and the PMD camera orientations are determined based on RGB images, therefore the true values are independent of the PMD image or range observations. In order to simplify further denotations, the following symbols will be used hereafter:

- [A]: Integrated calibration of range camera in joint setup with digital camera which is the proposed method of this study.
- [B]: Integrated calibration of a single range camera without additional digital camera, *i.e*., image internal camera parameters, external orientations and range error model are simultaneously estimated. However the RGB images are not utilized at this calibration scheme.
- [C]: Basic calibration of a single range camera excluding range observations equations and their corresponding error parameters, *i.e*., only intrinsic and external calibration of the intensity images of the range camera is performed.

In the following analysis, we will evaluate the results of our proposed calibration method, scheme [A], against schemes [B] and [C]. The residuals on image observations (*dx*,*dy*) and range measurements (*dρ*) of check images after calibration are reported in Table 5. The effect of intensity related error on range is also assessed. To do the task, range residuals are once assessed without considering intensity terms, (*c_6_–c_8_*), and once by taking these terms into account.

As an improvement of our method, scheme [A], over schemes [B] and [C] can be investigated as the percentage of reduction in RMS of range error and image coordinate residuals after calibration by each method. Table 6 indicates the effectiveness of our method from the independent accuracy assessments.

**Figure 7.**
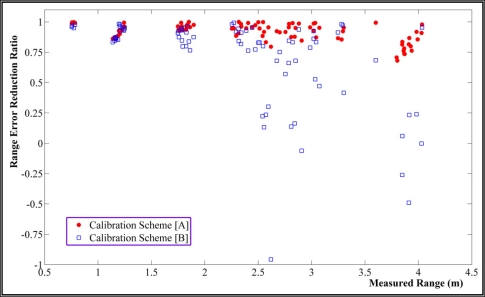
Range error reduction ratio provided by calibration schemes [A] and [B].

Figure 7 indicates the range error reduction ratios achieved by our calibration method, scheme [A], and compares it with scheme [B]. The error reduction ratio is defined as the difference of residual after and before range calibration divided to the initial range error. Therefore reduction ratio of zero means no improvement has taken place by calibration while unit ratio means that range error is totally eliminated and negative ratio indicates degradation. These results are obtained by considering intensity related error terms. It can be realized that the proposed calibration method of this study noticeably improves correction of range systematic errors in comparison with single integrated calibration of PMD camera, especially in ranges longer than two meters.

As discussed in [21], inclusion of range observations in bundle adjustment model increases the numerical stability of calibration. This stems from the fact that adding relevant constraints or additional observations ensures stable solutions to additional parameters. In the case of the range camera, a planar test field is used for range calibration to avoid scattering and multi-path errors. However, locating object points on the same plane causes a great correlation between internal and external camera parameters; especially *x_p_*, *y_p_* and *f* would be strongly correlated to the *X*, *Y* and *Z* coordinates of perspective center, respectively. Adding range observations to bundle adjustment equations provides extra information on imaging scale, thus reducing the correlation of these parameters [33]. However, the small field of view (FoV) of the PMD camera increases ambiguity between focal length, depth deviation and camera pose, which leads to unstable pose estimation results [14]. This problem is overcome with our joint calibration method. The fact is that in the joint calibration method, 2D digital images handle most of the PMD camera pose estimation. This kind of integrated bundle adjustment for PMD camera calibration provides the conditions similar to the calibration of a normal high resolution camera with wide FoV in a stable network along with additional range observations.

The correlation coefficient between parameters *X_i_* and *X_j_* is computed from variance and covariance values in the covariance matrix of unknown parameters from the bundle adjustment, $\sum_{\hat{\vec{X}}}$, by the following equation:

(26) $$\mathrm{corr}\left(X_{i},X_{j}\right)=\frac{\sum_{\hat{\vec{X}}}\left(i,j\right)}{\sqrt{\sum_{\hat{\vec{X}}}\left(i,i\right).\sum_{\hat{\vec{X}}}\left(j,j\right)}}$$

**Figure 8.**
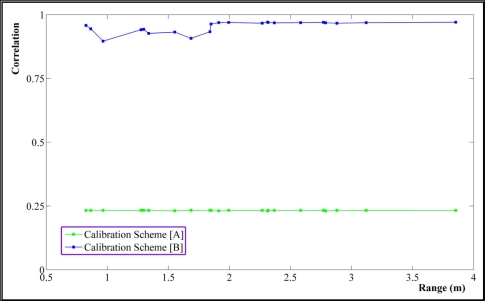
Correlations between principal distance and perspective centre of PMD camera *vs.* range.

The correlation value of 0 means two parameters are fully de-correlated while 1 means they are thoroughly correlated. Figure 8 shows great reduction, almost 75%, in correlations between principal distance and perspective center (PC) of the range camera after applying our calibration method in comparison with scheme [B].

**Figure 9.**
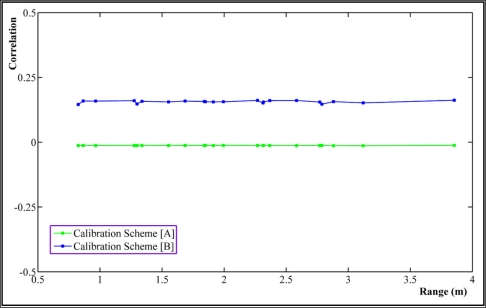
Correlations between rangefinder offset and perspective centre of PMD camera *vs.* range.

Other concerning dependencies may occur between the rangefinder offset (*c_0_*) and PC as well as the rangefinder offset and the principal distance [21]. The former correlations are depicted in Figure 9. The average value of the correlation between *c_0_* and PC is 0.16 by calibration scheme [B], which is reduced to −0.013 by our calibration approach. Regarding the latter concern, the correlation between *c_0_* and the principal distance is 0.1558 from calibration scheme [B] which is improved to −0.0027 by the proposed calibration method [A].

There also exists an average correlation of −0.0816 between the parameter *c_1_* and the perspective center of the range camera by the approach [B], which is itself a small value. However the mentioned correlation is improved to 0.0002 by the calibration scheme [A].

According to [23], the correlation between the distance related error terms and the rangefinder offset is concerning, especially in an integrated approach. The largest correlation detected among calibration parameters is between the rangefinder offset, *c_0_*, and parameter *c_1_*; −0.95 by calibration scheme [B], which is reduced to −0.71 by the proposed approach [A].

## 6. Conclusions

ToF cameras, based on PMD technology, provide range images at high frame rates which can be a valuable 3D data source for many applications. The errors associated with such measurements cannot be fully eliminated, but can be reduced to some extent by means of appropriate calibration procedures.

In this paper the integrated self-calibration of a range camera via a joint setup with a digital camera has been proposed and evaluated. The self-calibration bundle adjustment is performed based on observation equations of image point coordinates on intensity images of both cameras as well as range measurements of the PMD camera. The calibration results are improved by the presented approach regarding that:

- Most pre-researched systematic error sources affecting the range accuracy are taken into account,
- Image and range observations are adjusted in an integrated bundle adjustment,
- Problems coherent to small FoV of range camera such as high correlation between interior and exterior orientation parameters are overcome, and
- Specific equipment such as robot arms or laser scanners are not required.

Based on independent accuracy assessments, the proposed method achieved an average improvement of 83% in RMS of range error and 72% in RMS of coordinate residuals, over that achieved with basic calibration, *i.e.*, calibration of a single PMD camera excluding range observation equations. The method also introduced 25% and 36% improvement on RMS of range error and coordinate residuals respectively, over that obtained by integrated calibration of a single PMD camera. The joint system of range and RGB camera, calibrated by the approach of this article, is intended to be utilized as imaging sensor of a mobile mapping system.

## Figures and Tables

**Table 1.** PMD[vision]-O3 camera specifications.

**Sensor Array Size (pixels)**	64 (v) × 48 (h)	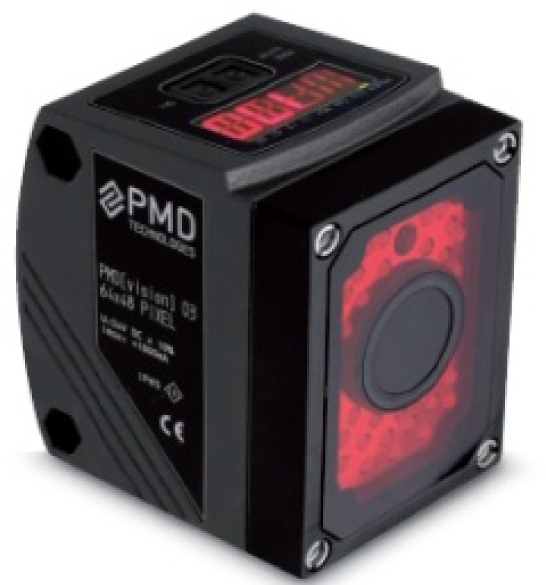
**Field of View (°)**	40 (v) × 30 (h)	
**Internal Illumination (nm)**	850	
**Working Range (m)**	0.2–4	
**Maximum Sampling Rate (Hz)**	25	
**Pixel Size (mm)**	0.1	

**Table 2.** Canon Power Shot SX1 IS camera specifications.

**Sensor Array Size (pixels)**	1,080 (v) × 1,920 (h)	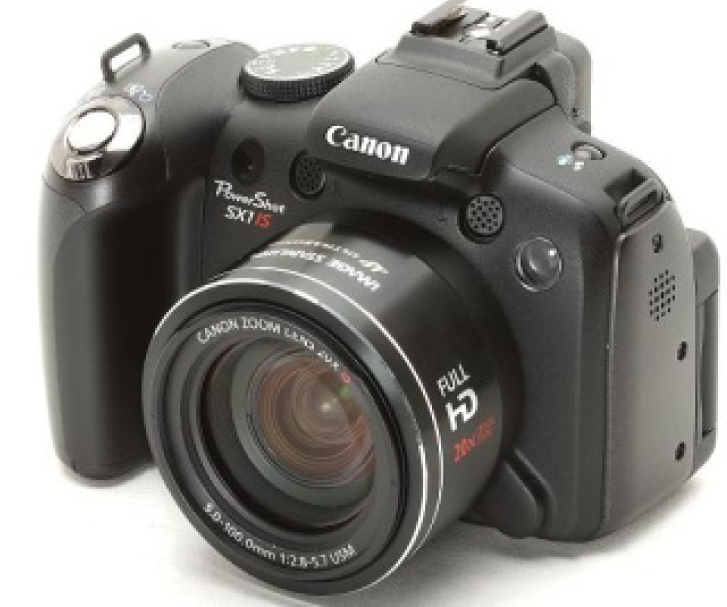
**Pixel Size (mm)**	0.005	
**Sensor Type**	CMOS	
**Nominal Focal Length (mm)**	Set to 5	
**Shutter Speed (sec)**	Set to 1/125	
**ISO Speed**	Set to 400	

**Table 3.** Digital camera parameters from pre-calibration process.

**Parameter**	**Estimated Value**	**Standard Deviation**
*K_1_*	0.003658	0.000006
*K_2_*	−0.000057	0.000000
*P_1_*	−0.000215	0.000005
*P_2_*	0.000141	0.000005
*x_p_* (mm)	−0.054075	0.000266
*y_p_* (mm)	0.084998	0.000208
*f* (mm)	7.536374	0.001152
*A_1_*	−0.007454	0.000023
*B_1_*	−0.000147	0.000022

**Table 4.** Range camera calibration parameters from integrated joint calibration.

**Parameter**	**Estimated Value**	**Standard Deviation**	**Parameter**	**Estimated Value**	**Standard Deviation**
*K_1_*	−0.027688	0.001897	*C_2_*	−0.022743	0.003890
*K_2_*	0.007355	0.000137	*C_3_*	0.003958	0.000078
*K_3_*	−0.000693	0.000169	*C_4_*	0.000074	0.000000
*K_4_*	0.000022	0.000007	*C_5_*	−0.000072	0.000000
*P_1_*	0.000635	0.000017	*C_6_*	0.003516	0.000985
*P_2_*	0.000627	0.000016	*C_7_*	−0.000038	0.000000
*x_p_* (mm)	0.017057	0.001764	*C_8_*	0.000000	0.000000
*y_p_* (mm)	0.072515	0.001738	*ω_2_* (rad)	0.011210	0.004765
*f* (mm)	8.041601	0.001563	*φ_2_* (rad)	0.083192	0.00547
*A_1_*	0.000785	0.000355	*κ_2_* (rad)	0.001816	0.000535
*B_1_*	0.001542	0.000317	*ΔX* (m)	0.183448	0.000731
*C_0_*(m)	−0.120160	0.008466	*ΔY* (m)	−0.015591	0.000492
*C_1_*	0.032551	0.017607	*ΔZ* (m)	0.017933	0.000265

**Table 5.** Residuals on observations with calibration schemes [A], [B] and [C].

**Calibration Scheme**	**A**			**B**			**C**		
**Residual**	**Mean**	**STD**	**RMS**	**Mean**	**STD**	**RMS**	**Mean**	**STD**	**RMS**
*dx* (μm)	8.0	6.0	8.6	10.4	9.7	14.2	27.4	18.3	32.9
*dy* (μm)	9.1	7.6	10.1	12.2	8.7	15.0	27.6	18.1	33.0
*dρ* (mm) [With Intensity]	8.844	6.514	10.963	12.147	8.212	14.637	56.582	32.380	65.102
*dρ* (mm) [Without Intensity]	13.067	10.482	16.715	12.764	10.257	16.339			

**Table 6.** Improvements achieved by the proposed calibration method.

	**Improvement of scheme [A] over [C]**	**Improvement of scheme [A] over [B]**
*RMS of Image Coordinates Residual*	71.6%	36.0%
*RMS of Residual on Range*	83.2%	25.1%
